# Bad Feelings, Best Explanations: In Defence of the Propitiousness Theory of the Low Mood System

**DOI:** 10.1007/s10670-023-00773-5

**Published:** 2024-01-22

**Authors:** James Turner

**Affiliations:** Department of Philosophy, University of Sheffield, 45 Victoria Street, Sheffield, S3 7QB UK

**Keywords:** Low mood, Depression, Proper function, Evolution, Adaptation

## Abstract

There are three main accounts of the proper function of the low mood system (LMS): the social risk theory, the disease theory, and the propitiousness theory. Adjudicating between these accounts has proven difficult, as there is little agreement in the literature about what a theory of the LMS’s proper function is supposed to explain. In this article, drawing upon influential work on the evolution of *other* affective systems, such as the disgust system and the fear system, I argue that a theory of the proper function of the low mood system should: (i) account for the reliable, distal causes and effects of the system’s activation, and (ii) explain how having a system that performed such a function increased fitness in ancestral environments. On this basis, I show that the proper function of the low mood system is to limit resource expenditure in relatively unpropitious circumstances, exactly as hypothesised by the propitiousness theory.

## Introduction

Evolutionary theorists are interested in discovering the *proper function* of various adaptations in organisms—they are interested in answering the question of what certain traits/systems were selected for (by natural selection) (Garson, 2019). There are several reasons for this interest. Not only does knowing a system’s proper function help us understand why that system *behaves in a certain way* (McClamrock, 1991), it also explains the *very existence* of that system (Garson, 2019), and allows us to make sense of the system’s *normativity* (*ibid*)—e.g., pancreases don’t just make insulin; they’re *supposed* to make insulin (Khin et al., 2023). And, finally, when it comes to psychological systems, many philosophers believe that knowing a system’s proper function can shed light on the *intentional content* of the mental states that the system tokens (see Schulte & Neander, 2022 for a review of the literature).

Many scholars have now put forward convincing accounts of the proper function of the systems underlying affective capacities such as disgust (Curtis et al., 2011), anxiety (Price, 2003), and pain (Broom, 2001). Much work has also been done to try to discover the proper function of the *low mood system* (LMS) (Allen & Badcock, 2003; Kinney & Tanaka, 2009; Nesse, 2019). What’s the point of having such a system? Why did evolution give us the capacity to experience low mood states? Answering these questions is not only of interest for the aforementioned reasons, but also because it can pave the way for more effective treatments of low mood disorders (Nesse, 2019; Schroder et al., 2023).

However, answering these questions has proven difficult. It’s not that evolutionary theorists have no candidate answers; rather, they have too many. In fact, there are at least three major theories of the proper function of the LMS (all of which see a great deal of support in the contemporary literature): the *social risk theory* (Allen & Badcock, 2003; Constant et al., 2021), the *disease theory* (Doyle et al., 2019; Kinney & Tanaka, 2009), and the *propitiousness theory* (Nesse, 2000, 2019).^1^ The aim of this article is to put this debate to an end: there are strong reasons, I maintain, to favour the propitiousness theory over its rivals.

Before I can begin my argument, however, we must first understand why none of these theories have become the received view. I contend that one of the key reasons is that evolutionary theorists working on the LMS lack an agreed-upon framework by which to judge a theory. This is highlighted by the fact that defenders of these different theories tend to focus on different data/results. For example, Allen and Badcock (2003) defend the social risk theory by pointing out that depression is associated with heightened amygdala activity, and that the amygdala has been shown to play an important role in complex social judgements. In contrast, supporters of the propitiousness theory typically ignore this type of neuroscientific evidence, and instead extensively consider clinicians’ observations of behavioural and cognitive changes in depressed individuals (Nesse, 2000, 2019).

In order to break this long-standing stalemate, in this article I propose that we should adopt the framework used by evolutionary theorists working on *other* affective systems (Curtis et al., 2011; Kavaliers & Choleris, 2001; Price, 2003), according to which a theory of the proper function of an affective system should:

(i) account for the reliable, distal causes and effects of the system’s activation;
(ii) explain how having a system that performed that function increased fitness in ancestral environments.

These explananda, I will argue, are both *core* and *minimal*. ‘Minimal’ in the sense that any evolutionary theorist, regardless of their theoretical inclinations and background, would expect a theory of an affective system’s proper function to account for them; ‘core’ in the sense that any evolutionary theorist should reject a theory that fails to account for them, and should have a strong reason to accept a theory that accounts for them.

I will further argue—and this is the most important contribution of this article—that when we apply this framework, it should become clear that the proper function of the LMS is to limit resource expenditure in relatively unpropitious circumstances, as only this hypothesis can satisfy both minimal, core explananda. Given that this hypothesis is exactly that put forward by the propitiousness theory, this article thus constitutes a defence of this approach over its rivals: the proper function of the LMS is not to help organisms minimise risk in social environments, as per the social risk theory, nor is it to promote disease avoidance/recovery, as per the disease theory, but it is to limit resource expenditure when there is less chance of making gains (e.g., climbing social hierarchies, improving interpersonal bonds, gathering food, etc.) that are worth the resource investments (e.g., spent energy, eaten food, sacrificed social bonds, etc.) than normal.

## Points of Agreement

The fact that disagreement looms large among evolutionary theorists working on the LMS doesn’t mean that they disagree on everything. In fact, there are three points of agreement that are important to present from the outset since they will play a key role in the discussion to follow.

### A Unified LMS

The first point of agreement is that there is *one, unified* LMS—all these authors are concerned with *the* low mood system (Allen & Badcock, 2003; Andrews and Thomson Jr. 2009; Kinney & Tanaka, 2009; Nesse, 2000, 2006). Since one might instead think that there are *multiple* low mood system*s*, let me briefly consider some of the reasons given in the literature for this claim, and provide you with one of my own.^2^

Firstly, Nesse (2019) notes that low mood feels like one thing, that we have a word for it, and that we can recognise descriptions of low mood in others. Why does this give us reason to think that the LMS is a unified system? Affective states that are the activations of different systems (e.g., fear and disgust) have different phenomenologies, while affective states that are the activations of the same system (e.g., different instances of fear) share a common phenomenology. As mentioned, from a first-person perspective, low mood feels like one thing, and most people indeed recognise descriptions of low mood with ease. Phenomenological evidence thus supports the hypothesis that low mood is the activation of a single system.

Of course, it is clearly true that people *experience different things* while in a low mood. For example, if I’m in a low mood because I’m ill, I will have a different *overall* experience than if I was in a low mood following, say, a social failure. The best explanation of this phenomenon, however, is not that there are two types of “low mood phenomenologies”, i.e., the illness-low-mood-quale and the social-failure-low-mood-quale. Rather, it is that while these two instances of low mood share a *core* phenomenology—some sense of feeling low/depressed (descriptions of which allow us to identify low mood in others)—each of them is accompanied by *further* distinct feelings (for example, when ill, one will experience aches, pains, feelings of swelling, or a general feeling of being sick, but these feelings aren’t likely to be present after social failure). Let me also point out that this idea of core phenomenology is supported by experimental evidence (Nummenmaa et al., 2014).

Secondly, evolutionary theorists think that there is one LMS because low mood’s symptoms are highly correlated, and there isn’t any symptom that is associated with some low-mood-eliciting circumstances but not others (Keller & Nesse, 2005; Nesse, 2019; Nettle & Bateson, 2012). We can unpack this argument as follows. Consider fear. We think there is a single fear system because even though fear causes us to do different things when confronted with different situations—I might try to squash a spider, but I will flee when confronted with a bear—the huge overlap in symptoms (increased heart rate, excess sweating, etc.) tells against the idea of separate fear systems. The same principle applies to the LMS.

Thirdly, Nesse (2006) points out that positing that there is a unified low mood system implies that different drugs that act on different parts of the system should result in a general improvement in mood. This is because if there were multiple low mood systems, it should be possible to selectively inhibit them. However, pharmacological interventions decrease low mood across the board, and they do so regardless of their specific neurochemical pathway (Harmer et al., 2017), thus supporting the view that there is one LMS.

Finally, I add that evidence for their being a unified LMS also comes from data on hypomania. During hypomanic states, those with hypomania are unable to go into a low mood, regardless of the low-mood-inducing stimuli they receive (Doran, 2008). In contrast, it doesn’t seem that there are any conditions in which people are capable of responding to some stimuli, but not to others, with low mood. This lack of selectivity again speaks in favour of a single LMS, as if there were more systems, we would expect there to be conditions that manifest specifically when these systems break down.

### The LMS’s Outputs

If the first point of agreement had to do with there being one system underpinning low mood, the second point concerns the outputs of this system: evolutionary theorists agree that the LMS is the system responsible for tokening both mild low mood and depression (Allen & Badcock, 2003; Kinney & Tanaka, 2009; Nesse, 2019).^3^ The reasons why they think this are as follows. First, these mental states have all the same symptoms—feeling ‘down’, fatigue, psychomotor retardation, altered appetite, a lack of interest and motivation in everyday activities, anhedonia (a diminished ability to feel pleasure), pessimism, self-reproach, and a general, diminished ability to think (Nettle, 2009). Second, there is much overlap in the neural underpinnings of depression and experimentally induced mild low mood (Andrews & Thomson, 2009).

This doesn’t mean that there isn’t a difference between mild low mood and depression. The difference, however, is a difference of degree rather than of kind: to be considered depressed, one must exhibit many low mood symptoms at the same time, and these symptoms must last for more than two weeks and cause significant disruption to one’s normal, everyday activities (American Psychiatric Association 2013). Since both mild low mood and depression are considered as activations of the LMS, I will use the term ‘low mood’ as a catch-all term for activations of the LMS.

It should be noted, however, that while the LMS is supposed to output both mild low mood and depression, it is not hypothesised to underpin *any* negative feeling whatsoever (Allen & Badcock, 2003; Kinney & Tanaka, 2009; Nesse, 2019). Pains, negative emotions, and other moods such as anxiety or irritation are not thought to be tokened by the LMS—only those states that are characterised by the above symptoms are.

### Defining ‘Proper Function’

And now to the last point of agreement. The aim of this paper, as you know, is to give an account of the proper function of the LMS. Although there are several accounts of proper function in the philosophical literature (see Christie et al., 2021), all people working on the proper function of the LMS, in fact all people working on the proper function of affective systems (e.g., Curtis et al., 2011; Kavaliers & Choleris, 2001; Price, 2003), adopt the following definition of proper function: the proper function of a system is what that system did that led to it being selected for by natural selection. I too adopt this definition.

Agreement, however, ends just about here. While everyone agrees that there is one LMS that tokens both low mood and depression (but not other negative feelings), and while everyone agrees about what it means to say that we want to discover the LMS’s proper function, disagreement looms large regarding how to discover it. It is to this issue that I now turn.

## The Minimal, Core Explananda

### How to Discover Proper Functions

The activity of the LMS correlates with many variables. As mentioned in the Introduction, this has led evolutionary theorists with different scientific backgrounds to focus on differing sets of data when formulating and providing evidence for their theories. For instance, some researchers focus on explaining the link between low mood and physical disease (Kinney & Tanaka, 2009), while others attempt to account for the role of low mood in status competition (Price & Sloman, 1987); some draw on data on the neural underpinnings of low mood (Allen & Badcock, 2003), while others ignore these data altogether (Nesse, 2000) and instead resort to first-hand clinical observations to support their theory (Nesse, 2019).

To remedy this situation, we need to develop an agreed-upon framework by which to judge competing theories. I propose we model the inquiry of the proper function of the LMS on the approach used by evolutionary theorists working on other affective systems, such as fear (Kavaliers & Choleris, 2001) and disgust (Curtis et al., 2011). These researchers were able to identify a set of what I argue are *minimal, core explananda*. These explananda are *minimal* in the sense that any evolutionary theorist, regardless of their theoretical inclinations and background, would expect a theory of an affective system’s proper function to answer them. Moreover, these explananda are also *core* in the sense that any evolutionary theorist should reject a theory that fails to answer them, and should have a strong reason to accept a theory that accounts for them. Here they are. A theory of the proper function of an affective system should (Curtis et al., 2011; Kavaliers & Choleris, 2001; Price, 2003):

(i) account for the reliable, distal causes and effects of the system’s activation;
(ii) explain how having a system that performed that function increased fitness in ancestral environments.

I will now unpack and defend this proposal.

### Causes/Effects

First, let’s clarify (i). Why are we talking about systems’ *activations*? Affective systems are *facultative adaptations*—they are only useful, and therefore only activate, in certain circumstances. E.g., fear (the activation of the fear system) is only useful when we are in danger, so we expect the fear system to only activate when danger is present (or there are indicators of danger). Therefore, if we want to know what a system was selected for, we need to look at cases where the system gets activated.

What do we mean by *reliable* and *distal* causes/effects? In the case of affective systems, *distal causes* tend to be either situations that pose certain adaptive challenges, or cues that indicate the presence of such situations. The fear system, for example, is typically activated in dangerous situations, or when encountering something that indicates that one’s situation is dangerous (e.g., when encountering a large creature) (Adolphs, 2013). Distal *effects* are typically *behavioural* responses that follow from a system’s activation. For example, the typical distal effects of fear are fighting, fleeing, and freezing (*ibid*). By contrast, an example of a *proximate* cause would be a particular neural firing that causes the system to activate, and a proximate effect would be pupil dilation. ‘*Reliable* causes/effects’ instead refers to the kinds of thing that typically, across a large number of individuals, are responsible for an affective system’s activation, and the types of effect that such an activation typically has across a wide range of individuals. For example, predators, high places, and the dark are a reliable cause of fear (*ibid*), while butterflies are not.

Why focus on *distal* causes/effects? The fear system is activated by both predators and neural activity in the amygdala, so why do (and why should) evolutionary theorists working on fear give pride of place to the former (Kavaliers & Choleris, 2001)? The reason is simple. To be selected for, a system must have increased organisms’ *fitness* (their ability to survive and reproduce) in ancestral environments (Spencer, 1864)—the environments in which the distant ancestors of modern organisms lived and had to adapt to. However, while it’s clear how a system that is causally sensitive to predators (or cues that indicate the presence of predators) *could* have enhanced fitness, it’s unclear what the evolutionary benefit of a system that is causally sensitive to activity in the amygdala *could* have been. The same applies, *mutatis mutandis*, to distal effects. For this reason, theories of proper function typically make *direct predictions* about distal causes/effects, whereas they don’t provide such *direct* predictions about proximate causes/effects.

Why focus on *reliable* causes/effects? If a system wasn’t reliably sensitive to certain cues, or didn’t reliably respond in a certain way, it’s unclear how that system could have increased fitness. E.g., if fear didn’t reliably arise in dangerous situations, it wouldn’t be able to help organisms avoid such situations. Accordingly, we should put aside fear’s unreliable causes/effects and focus on its reliable causes/effects, because we can only say with confidence that the latter are indicative of a *properly functioning* system. This is why evolutionary theorists are interested in explaining why the fear system is activated by predators, for example, but not in explaining why it’s sometimes activated by butterflies—for all we know, the latter activation might be indicative of a misfiring, and hence potentially *dysfunctional*, system (*Cf.* Kavaliers & Choleris, 2001).

### Fitness Enhancement

Let’s move onto (ii). We’ve already established that a theory of an affective system’s proper function shows how the system *could* improve fitness. However, one also needs to show that it *did* so. To reiterate: in order to have been selected for, a system *must* have increased organisms’ fitness in ancestral environments; therefore, explaining how an affective system increased fitness in such environments is non-negotiable. But how can one show that a system increased fitness in the *past*? There are a few options. If certain adaptive problems are still as relevant today as they were in the past, we can look to see if having a system whose activation has such causes and effects improves fitness in the present day. Alternatively, we could see if blocking a certain response has a *negative* impact on fitness. If the adaptive problem is no longer relevant in the present, we can create mathematical models that test whether having a certain system would improve fitness in environments that are as close to ancestral environments as possible. To be clear: these tests/models should provide evidence that a system improved fitness *in the way specified by the theory of proper function*. If the proposed proper function of fear is to avoid danger, then we must show that it increased fitness specifically by helping organisms avoid *danger*.

Finally, evidence for (i) and (ii) may come from animal models, provided that those animals exhibit relatively similar behaviours under similar circumstances. E.g., the fact that rats exhibit fight/flight/freeze responses to predators can be used as evidence for a theory of the proper function of the fear system. However, given the differences between humans and other animals, we shouldn’t draw our conclusions solely from animal models, but instead use them in *conjunction* with data from humans.

To clarify the above, and to elucidate how (i) and (ii) interact in the construction of a theory of an affective system’s proper function, let’s consider the case of disgust. The *pathogen avoidance theory* states that the proper function of the disgust system is to track and avoid pathogens (Curtis et al., 2011). As such, it predicts that disgust will be caused by situations where either pathogens are present, or where there are environmental indicators of pathogens, and that disgust will cause behaviours that aid in pathogen avoidance. These predictions are confirmed by data. The reliable, distal causes of disgust are things such as rotten foods, expelled bodily fluids, and open wounds, while its reliable, distal effects include behaviours like vomiting, spitting, and closing one’s nostrils (*ibid*). All these causes contain, or indicate the presence of, pathogens, and all these effects can help prevent pathogens from entering our bodies. Thus, this hypothesis satisfies explanandum (i). What about (ii)? Pathogen-avoidance is an adaptive problem that is as relevant today as it was in the ancestral past, and increased disgust sensitivity is associated with increased sensitivity to infections and a lower infection rate in current humans (Cepon-Robins et al., 2021)—the specific fitness benefit predicted by the pathogen-avoidance theory. Therefore, we have good reason to assume that the disgust system increased fitness in ancestral environments by helping organisms avoid infection and disease. Since the pathogen-avoidance theory explains both (i) and (ii), we have a strong reason to accept it. As it happens, the theory is now almost universally endorsed amongst evolutionary theorists (Oaten et al., 2009).

### Taking Stock

The problem: there is widespread disagreement about what a theory of the LMS’s proper function should explain. My solution: take inspiration from what evolutionary theorists have been doing regarding the proper function of *other* affective systems. Accordingly, I propose that a theory of the proper function of the LMS that doesn’t account for (i) and (ii) should be rejected, while we have excellent reason to endorse a theory that accounts for them. Now I will review the data on the reliable, distal causes/effects of the LMS’s activation.

## Causes and Effects: The Data

### Clarifications

First: how do we identify which states are *activations* of the LMS? As already pointed out, there is universal agreement among evolutionary theorists that both mild low mood and depression are activations of the LMS (Allen & Badcock, 2003; Kinney & Tanaka, 2009; Nesse, 2019), due to the fact that they have almost all the same symptoms (Nettle, 2009), and the fact that there is much overlap in the neural underpinnings of depression and experimentally induced mild low mood (Andrews & Thomson, 2009).

Please notice that since the presence of reliable, distal causes/effects is a hallmark of an adaptation, if such causes/effects exist in the case of depression, we should then consider the latter as an activation of a *properly functioning* LMS. This might generate a worry in the reader—given that perhaps the most influential theory of disorder, the Harmful Dysfunction Theory, has it that dysfunction is necessary for disorder (Wakefield, 1992), if depression is an activation of a properly functioning LMS, depression cannot be a disorder, and this flies in the face of depression’s classification as a mental disorder by diagnostic manuals such as the DSM-5 (American Psychiatric Association, 2013). But this worry is misplaced, since it has become widely accepted that most cases of depression are activations of a properly functioning LMS (Andrews & Thomson, 2009; British Psychological Society, 2020; Nesse, 2019).^4^^,^^5^ There are many reasons for this acceptance. Firstly, we have little reason to think that most cases of depression are activations of a *dysfunctional* LMS. The DSM diagnoses depression based on the severity and duration of symptoms, which is a poor indicator of dysfunction (Nesse, 2019). For example, pain may be extreme and long lasting, but if it’s in response to a severe and persistent injury, then there is nothing dysfunctional about the pain system. Moreover, depression rates are highest in early reproductive years, and this is unlike most dysfunctions/diseases, which typically become more common as people age (Keller & Nesse, 2005). Finally, as mentioned, reliable, distal causes/effects are hallmarks of adaptations. Since the following data show that depression has reliable, distal causes/effects, then the data themselves serve as further indication that most cases of depression are activations of a properly functioning LMS. Now, onto low mood’s reliable, distal causes/effects.

### Reliable, Distal Causes

The most important thing to note about the distal causes of mild low mood and depression is their domain-generality (Keller & Nesse, 2005; Kessler, 1997). Admittedly, over 80% of cases of depression are precipitated by severe, negative life events (Brown and Harris 2001), but these events do not all fit into one particular domain. Here are the main triggers for mild low mood and/or depression.

*Social rank losses/failures*: Cases of depression often arise after major losses in social rank (e.g., the loss of a job) (Kessler, 1997). Repeated failure to succeed at work has also been shown to lower mood and raise the risk of depression (*ibid*). Moreover, repeated work-related failures have been shown to reliably prolong the duration of depressive episodes, while success that counteract these failures (such as getting a promotion after a previous, failed attempt) have been shown to reliably reduce the duration of people’s depression (*ibid*).

*Interpersonal losses/failures*: Major losses of interpersonal relationships, such as the death of a loved one or the separation from a partner, are common causes of depression (Keller & Nesse, 2005). Like with social rank losses/failures, repeated, smaller failures to engage in successful interpersonal and romantic relationships have been shown to lower mood and raise the risk of depression (and prolong current depressive episodes), and successes that counteract these losses/failures (such as entering into a new relationship after a breakup) reliably reduce the duration of people’s depression and improve mood (Kessler, 1997).

*Personal failures:* Repeated failure to achieve personal goals, both concrete (e.g., exercising more or creating a piece of art), and abstract (e.g., getting closer to God), lowers mood and increases the risk of depression (Moberly & Watkins, 2010; Street, 2002). Moreover, breaking down personal goals into smaller, more manageable goals (and thus increasing the rate at which one succeeds in fulfilling personal goals) has been shown to increase mood and reduce the duration of people’s depression (J. S. Beck, 2011).

*Slow progress towards goals*: As an addendum to the above, it has been shown that slower than expected progress towards a goal (social, interpersonal, or personal) reliably lowers mood. In fact, mild low mood is affected more by the rate of progress towards a goal than by an individual failure (Lawrence et al., 2002).

*Illness*: There is evidence that illness and ill health can cause depression. Many studies have found a correlation between inflammatory cytokines and other biomarkers of disease and depression (Miller & Raison, 2016), and there is evidence, albeit thin evidence, that some anti-inflammatory drugs can be useful in the treatment of depression (Köhler-Forsberg et al., 2019). People also often become depressed following serious injury or illness (Goodwin, 2006). Moreover, exercise and heathy eating have both been shown to improve mood and assuage low mood’s symptoms (Craft & Perna, 2004; Ljungberg et al., 2020), suggesting that ill health is instrumental in causing low mood.

### Reliable, Distal Effects

So, low mood is reliably caused by many different types of elicitors. But what of its effects? I now show that both mild low mood and depression have reliable and *general* effects on behaviour. More precisely, low mood has global demotivational effects—the LMS, when activated, reliably demotivates social, interpersonal, and personal behaviours *all at once*, and *no matter the cause*.

*Socialisation:* Depressed individuals typically socialise less than non-depressed individuals and are less skilled at social interactions (Gotlib, 1992). These data are correlative, but upon further analysis, we can infer that depression causes people to socialise less than non-depressed individuals. Firstly, depression often figures into causal explanations of such behaviour (Rossi, 2019). It’s not uncommon to hear things like “I didn’t go to the party because I was too depressed”. Secondly, and more importantly, various antidepressants have been shown to increase sociability of depressed individuals (Briley & Moret, 2010), indicating that reduced socialisation causally depends upon depression.

*Work:* Depressed individuals tend to work less than non-depressed individuals (Lerner & Henke, 2008). These data are also correlative, but we can infer that depression causes people to work less than non-depressed individuals with further analysis. Therapies that alleviate depression and depressive symptoms have been shown to have a positive impact on work performance (Sledge & Lazar, 2014), indicating that depression is causally responsible for diminished working.

*Parenting:* Depressed individuals parent less, on average, than non-depressed individuals, resulting in an overall negative impact on the parent–child relationship (C. T. Beck, 1995). Again, these data are correlative, but one experiment has showed that treating mothers’ depression with therapy improves mother–child relationships (Murray et al., 2003), and a review of the literature shows that treating mothers’ depression generally improves children’s functioning (Gunlicks & Weissman, 2008), suggesting that depression is the cause of these negative effects.

*Non-Social Behaviours:* Low mood has a complicated effect on non-social activities, such as creating art, or going for a walk by oneself. Low mood is, in general, negatively correlated with behavioural activation (i.e., seeking/wanting behaviours), across *social* and *non-social* domains (Dickson et al., 2017). Moreover, many severely depressed individuals struggle to do just about anything, even small tasks like getting out of bed (Kanter et al., 2008). However, while some data suggest that low mood generally lowers people’s participation in leisure activities (both social and non-social) (Nimrod et al., 2012), other data suggest that depressed individuals partake in at least more sedentary hobbies, such as watching television, than controls, and may even exercise more (though the latter is debated) (Blanco & Barnett, 2014).

What should be made of this? Firstly, it should be noted that some depressed individuals use sedentary hobbies as coping mechanisms, and may exercise as a means of assuaging low mood symptoms (Nimrod et al., 2012). Secondly, depressed individuals report less willingness to engage in, and gain less enjoyment from, many of the activities that they participate in (Blanco & Barnett, 2014). This is consistent with the fact that approximately 70% of depressed individuals experience anhedonia (Shankman et al., 2014). A likely explanation of these data, then, is that low mood demotivates engagement in non-social activities, but that some people engage in these activities despite their low mood, typically as a coping mechanism.

We have even more reason to think that low mood demotivates non-social behaviours when we look at mouse experiments. Mice with induced low mood symptoms forage less (for themselves) than control mice (Yang et al., 2014). Moreover, mice treated with antidepressants (and who therefore exhibit fewer low mood symptoms) swim for longer than mice that exhibit low mood symptoms in forced swimming trials—trials in which mice are put into an inescapable vat of water and timed to see how long they swim for before they stop and float instead (Can et al., 2012). This suggests that low mood affects basic survival behaviours, as mice swim in an attempt to escape the water and avoid drowning (though it should be noted that there is evidence that floating is actually the optimal strategy, as it helps mice conserve energy, thus lessening the chance of them drowning) (Molendijk & de Kloet, 2019).

*Hygiene.* Depressed individuals, on average, have poorer hygiene than non-depressed individuals. Specifically, they tend to engage in fewer hygiene behaviours, such as hand-washing (Slekiene & Mosler, 2017). We can infer that depression causes this lack of hygiene behaviours when we consider that people often report that their low mood *demotivates* them from engaging in behaviours such as washing/showering (Nimrod et al., 2012).

*Risk-Taking.* If low mood demotivated action and behaviour, we would expect depressed people to take fewer risks as a result of them partaking in fewer activities across the board. As it turns out, depressed individuals do take fewer risks, on average, in almost all domains (Yuen & Lee, 2003).^6^ Data on hypomania support the hypothesis that low mood is the cause of low risk-taking (Nesse, 2009). Hypomanics exhibit low mood symptoms and low risk-taking, *but not while hypomanic*. During hypomanic phases, they instead take risks like excessive spending, drunk-driving, and arguing with friends and family, which can lead to divorce, bankruptcy, and imprisonment (Doran, 2008).

### Looking Ahead

Low mood’s reliable, distal causes and effects are not limited to any one domain—low mood is caused by social, interpersonal, and personal losses/failures, as well as slow progress towards goals and poor health, and it has a *global* demotivational effect on behaviour, *regardless of cause*. A theory of the LMS’s proper function should be able to account for this. Furthermore, such a theory should provide evidence that the LMS increased fitness in ancestral environments. Naturally, each theory will provide a different explanation. In the following sections, I outline the three most popular theories of the LMS’s proper function: the *social risk theory* (Sect. 5), the *disease theory* (Sect. 6), and the *propitiousness theory* (Sect. 7), and argue that only the propitiousness theory can successfully account for (i) and (ii).

## The Social Risk Theory

The social risk theory states that the function of the LMS is to help organisms minimise risk in social environments (Allen & Badcock, 2003). When an individual’s ratio of social value vs social burden is critically low—i.e., when an individual becomes more of a hindrance than a help to their social community—the LMS activates in order to: (a) make one hyper-sensitive to social threats; (b) send signals that reduce social risk (e.g., submissive behaviours); (c) inhibit the climbing of social hierarchies and other risky social behaviours (e.g., behaviours that result in a competition for resources) (*ibid*). By bringing about (a)-(c), the LMS helps an individual minimising social risk-taking in socially risky circumstances (but not in safer circumstances).

A recent mathematical model of the social risk theory—in which instances of low mood are characterised by social withdrawal—has shown that such a strategy would be fitness-enhancing, specifically by reducing social risk (Constant et al., 2021), thus satisfying explanandum (ii). But how does the social risk theory comport with explanandum (i)? Not well; it has problems in explaining the non-social causes (Sect. 5.1) and effects (Sect. 5.2) of low moods.

### Reliable, Distal Causes

The social risk theory predicts that low mood will be caused by situations in which one’s social risk is high, or following events that indicate increasing social risk. As predicted, many of the life events that bring about low mood are tied to social risk. Consider events such as job losses, divorce, and the death of family members: the first makes someone more of a social burden, while the second and the third result in the person having fewer social connections to fall back on. The social risk theory also does a good job of explaining why disease often causes depression. Diseases are often debilitating and contagious, thus making a diseased individual a social burden.

This is all well and good, but the distal causes of low mood extend far beyond the social domain. Failure to achieve (or slow progress towards) personal goals, such as completing artistic projects or exercising, causes low mood. How can the *social* risk theory account for this? Granted, our personal and social lives are intertwined in complex ways, so some personal goal failures may in fact have deleterious effects on one’s social status. For example, failure to beat your own personal best time while running may lower people’s estimation of you, should you share your results with your peers. There are, however, other personal goal-failures that lead to low mood but don’t seem to have anything to do with the minimisation of social risk. Consider failure to create a piece of art. Even if such a hobby were undertaken in private, failure to create the artwork would likely lower mood, despite no clear connection to social risk, thus casting at least some doubt on the social risk theory.^7^ A similar problem emerges when we look at the effects of low mood.

### Reliable, Distal Effects

The social risk theory predicts that low mood will cause behaviours that could minimise one’s social risk. Thus, the theory seems well placed to explain why low mood affects a wide range of social behaviours: causing people to socialise, parent, and work less than those in a neutral mood (Beck, 1995; Gotlib, 1992; Gunlicks & Weissman, 2008; Lerner & Henke, 2008; Sledge & Lazar, 2014). The theory can easily explain why low mood causes people to socialise less than controls: if an individual poses more of a social burden than they do a social asset, retracting from social events could be beneficial. This same rationale can be used to explain diminished parenting and work behaviours in individuals with low mood. In this respect, Hagen’s (2011) criticism of the theory is misplaced. He argues that the theory should predict that those who experience low mood will work and parent more in an attempt to make themselves more socially useful. In contrast, since the theory states that the function of the LMS is to decrease social risk-taking when an individual is likely to be a social burden, I argue it predicts retraction from *all* social situations, including working and parenting.

On the subject of risk-taking, the social risk theory does a good job of explaining the fact that low mood diminishes risk-taking in many domains, as most types of risk have consequences for one’s social standing. Finally, the theory also explains data on mice’s foraging. In such experiments, mice with induced low mood forage less than those in a neutral mood. Though they forage for themselves, they can be seen by other mice. As such, by limiting their foraging, they plausibly signal that they are not using up valuable resources, thus indicating that they are not a social burden.

The problem for the social risk theory is rather that it does not predict that low mood will have an effect on behaviours that do not affect one’s level of social risk, while data indicate that it does, both in human and in non-human animals. People engage less in, and get less enjoyment from, previously enjoyably activities. Though some of these activities are social, some, such as going for a walk, are clearly non-social, and so cannot be explained by the social risk theory.

This effect on non-social activities is also seen in mice. Low-mood-induced mice swim less than those who do not exhibit low mood symptoms in forced swimming trials. Mice that exhibit low mood symptoms while in the water stop swimming quicker than mice treated with antidepressants (whom therefore do not exhibit such low mood symptoms). In these cases, swimming and floating are examples of basic survival behaviours, not social behaviours. One might argue that floating also acts as a signal for help from conspecifics, and so is a social behaviour after all. However, there are two problems with this response. First, it’s not clear that mice actually engage in such pro-social rescuing behaviour (Ueno et al., 2019), so it’s unlikely that drowning mice would float to signal for help. Second, even if mice did engage in rescuing behaviours, a mouse that floats is a bigger social burden than one that swims, as it requires being rescued by its conspecifics, so low mood should not cause such behaviours. In fact, the social risk theory predicts the opposite behaviour. If mice did engage in rescuing behaviours, mice in a low mood should swim more to avoid being a social burden and decrease the risk of hostility or ostracization from conspecifics.

Much the same can be said about hygiene. While one might hypothesise that poor hygiene signals a kind of social submission or a need for help, poor hygiene elicits negative reactions and social rejection from conspecifics (van der Geest, 2015). Thus, it’s hard to see why the LMS would make people’s hygiene worse if its function was to minimise social risk. If anything, it would predict the opposite—that depressed individuals would have better hygiene in order to minimise social risk. Taking all the above into consideration, we have good reason to reject the social risk theory, as it fails to satisfy explanandum (i).

## The Disease Theory

The disease theory states that the LMS’s function is (a) to promote disease-avoidance; (b) to stop one from spreading disease to one’s conspecifics; and (c) to foster disease-recovery behaviour (Kinney & Tanaka, 2009). According to the theory, the LMS promotes (a) and (b) by demotivating social and sexual interactions with others. In this way, one is less liable to catch a disease and to spread it. As to (c), the theory has it that the LMS causes lethargy, which promotes rest, which in turn is essential in recovering from illness. Let’s now see how the theory fares with respect to our minimal, core explananda.

### Reliable, Distal Causes

The disease theory predicts that the LMS will activate in situations where the chance of infection is high, or when one is already ill, or when there are cues that indicate potential or occurrent infection. In Sect. 4.2, I presented data that show: a correlation between biomarkers of disease and depressive symptoms; the effectiveness of anti-inflammatory drugs in the treatment of depression (though recall that this evidence is thin); the causal role of illness and poor health in precipitating depression. Clearly, the disease theory can account for these data.

Does the disease theory do a good job of explaining the social and interpersonal causes of low mood? I say that it does. Interpersonal losses such as the death of a partner, parent, or friend means that an individual has fewer people to call upon should they need help while ill. By the same token, those with lower social rank will be more likely to be harmed by disease than those in higher ranks, as they will not have the same level of influence to garner support from their conspecifics, hence a mechanism to prevent them from catching the disease would be beneficial to them. This effect of social rank on vulnerability to disease is seen in both humans and primates—in humans, low social rank, and specifically a lack of money, can result in people having limited access to healthcare (Cockerham et al., 2017); in many primate species, higher-ranked individuals get groomed more than lower-ranked individuals, reducing their chance of being harmed by parasites (Thierry et al., 1990).

The disease theory also makes sense of personal failures, something that the social risk theory has trouble with. Personal failures tend to induce stress, and chronic stressors have been shown to reduce the efficacy of the immune system (Segerstrom & Miller, 2004). It thus makes sense for low mood to arise in cases of stress to prevent an already weakened immune system to have to fight a disease. I therefore conclude that this theory is well-placed to explain the various causes of low mood. However, I argue that it doesn’t explain a key effect of the LMS.

### Reliable, Distal Effects

The disease theory predicts that low mood will cause a wide range of behaviours that minimise the chance of catching or spreading disease, or aid in disease recovery. Therefore, if the disease theory were true, we would expect individuals experiencing low mood to socialise less in order to avoid spreading or catching disease, and we would expect them to engage in fewer non-social activities in order to preserve energy to fight off infection. We would also expect these individuals to work and parent less, as doing so will help curb the spread of disease and help recovery. By the same token, we would expect mice in a low mood to both forage and swim less than mice in a neutral mood, as rest helps recovery from infection. All these predictions are borne out by data.

There is, however, one crucial piece of data that the disease theory cannot explain. It predicts that low mood should cause people to be more hygienic, as staying clean is one of the best ways to avoid contracting disease. However, things are exactly the other way around: low mood makes people’s hygiene worse, not better. These data cast serious doubt on the disease theory. If the function of the LMS is to help avoid disease, then it should not cause people to behave in way that makes them *more* susceptible to disease.

### Evolutionary Irrelevance

I want to conclude my discussion of the disease theory by highlighting another problem: it’s not clear that the LMS would be evolutionary beneficial, and hence an adaptation, if its function were to detect and avoid disease. Let me be clear: to have *one* such system is clearly fitness-enhancing. My point is that we already have one such system: the disgust system. As mentioned, there are overwhelming reasons to think that disgust helps us track and avoid pathogens. New adaptations are costly to build and maintain, so they cannot come without a purpose. But what could be the point of having two systems doing the same job?

One might respond that since low mood is associated with an increased sensitivity to signals of disgust (Surguladze et al., 2010), the LMS functions to enhance the disgust system. However, encountering disgusting stimuli, facial expressions associated with disgust, or even the word ‘disgust’, prime people to respond to disgusting stimuli more quickly than those not primed by such stimuli (Neumann & Lozo, 2012). This suggests that the disgust system is capable of enhancing itself, or can at least be enhanced by systems other than the LMS, once again casting doubt on the idea that LMS offered any evolutionary benefit.

The disease theorist might argue instead that the LMS helps one to respond to situations that increase the risk of disease that the disgust is not sensitive to. For instance, perhaps the LMS helps organisms avoid disease in the wake of social losses, whereas the disgust system is not sensitive to such social inputs. However, if the function of the LMS were to detect social (or interpersonal, or personal) indicators of disease, then the theory would not predict that disease itself would be a cause of low mood.

In order to overcome these worries, the disease theorists would have to provide evidence that not only shows how a system with their proposed function would have improved fitness in ancestral environments by helping organisms avoid disease, but that also shows how it would have improved fitness given the existence of the disgust system. Without this evidence, we have good reason to reject the disease theory.

## The Propitiousness Theory

Finally, we turn to the propitiousness theory. In this section, I outline it (Sect. 7.1), show how it satisfies both minimal, core explananda (7.2), and respond to an influential objection (7.3). I conclude by saying that we should endorse the propitiousness theory as our theory of the proper function of LMS.

### The Theory

According to the propitiousness theory, the function of the LMS is to limit resource expenditure, primarily by demotivating action and promoting disengagement from activities, in *relatively unpropitious circumstances* (Nesse, 2019). To understand this theory, we need to understand the notion of ‘unpropitious circumstances’. Let’s start from their opposite, namely, *propitious* circumstances.

According to Randolph Nesse, the key proponent this theory, a propitious circumstance is one in which there is a high chance of making net gains*.*^8^*Gains* are things whose attainment will benefit the individual in one way or another—things such as climbing social hierarchies, improving interpersonal bonds, improving physical health, gathering food, fulfilling personal or artistic goals, etc. (*Ibid*). Resource *investments* are those things that the individual uses/sacrifices to make gains. The most obvious investment is energy, but other resources such as the sacrifice of interpersonal bonds, or eaten/shared food, can be considered as investments. *Net gains* are gains that are worth more than the resource investments.

A relatively unpropitious circumstance, then, is one in which there is less chance of making net gains than normal. The function of the LMS is to track and respond to these circumstances appropriately. To borrow an example from Nesse (2019), suppose you’re an early human and a herd of mastodon are migrating through your territory. You are healthy and part of a strong social group, and the mastodon are plenty. The situation is propitious, as attempts to hunt mastodon will likely result in you gaining food and other materials without expending too much energy or other resources. However, if you were ill, the situation will be relatively unpropitious, as your infirmity would lessen the chance of your hunting success and increase your resource expenditure, thus making it less likely for you to make net gains from a hunting expedition. Likewise, falling out with conspecifics would also lower your chances of making net gains, as you would not have as many social resources to use when hunting. In these kinds of relatively unpropitious circumstances, the LMS functions to reduce motivation and promote disengagement so that you don’t waste resources on tasks when there is a minimised chance of net gain.

### What the Theory Can Do

How does this theory fare when it comes to satisfying explanandum (i)? Let’s begin with low mood’s reliable, distal causes. The propitiousness theory predicts that the LMS will be activated in relatively unpropitious circumstances, or following cues that indicate decreasing propitiousness. The LMS is activated by a wide variety of negative life events: social, interpersonal, and personal losses/failures, as well as ill health. This variety of causes is predicted by the propitiousness theory, as an event can become less propitious due to a number of different reasons. For example, illness makes making net gains more difficult as it makes us slower and increases energy expenditure, while social and interpersonal losses result in us having fewer social resources to draw upon when engaging in activities, and personal failures are evidence that what we thought we could achieve with the resources we have, we in fact cannot. As such, it’s no surprise that low mood is caused by such a wide variety of negative life events.

What about low mood’s reliable, distal effects? The propitiousness theory predicts that low mood will have a *global*, demotivational effect on behaviour in order to limit resource expenditure. After all, engaging in *any* activity uses resources—e.g., running uses energy, and socialising uses not only energy, but could also require one to share food or spend money. So, if the LMS’s proper function is to *generally* limit resource expenditure, we should expect individuals experiencing low mood to be less motivated to partake in activities in just about every domain, regardless of what specifically caused their low mood. This prediction is borne out in the data. Individuals in a low mood socialise, parent, and work less than those in a neutral mood.

Moreover, this theory explains why these individuals engage in fewer personal-goal activities, something that the social risk theory struggles to account for. Mice should swim and forage less as a product of the general, demotivational effect of low mood, and risk-taking should be generally reduced as a product of those in a low mood taking part in fewer activities across the board. What’s more, the propitiousness theory explains why those in severe low moods struggle to do just about *anything*—if the LMS has the proper function of generally demotivating behaviour to conserve resources, extreme low mood should demotivate people from engaging in just about *all* activities in order to conserve as many resources as possible.

The propitiousness theory also has serious advantage over the disease theory. Since the function of the LMS is *not* to aid in disease avoidance, we shouldn’t expect individuals in a low mood to have a better hygiene than controls. In fact, we should expect the opposite, given that the LMS’s function is to limit resource expenditure, and maintaining one’s hygiene requires energy (a resource).

Finally, the propitiousness theory offers both a simple and plausible model of how the LMS increased fitness in ancestral environments: limiting energy expenditure not only helps organisms make more net gains in the long run, but could also save them from wasting *all* their resources on unattainable outcomes. This is not a just-so story either. Nesse has modelled three strategies to resource investment, one that always invests a set amount of resources, one that always invests 10% of its resources, and one that limits its resource expenditure following failures (low mood), and invests more after successes (high mood) (Nesse, 2019). The results of the model show that as long as the environment is moderately predictable—i.e., failures more often than not follow failures, and success more often than not follow successes—the strategy that varies expenditure (the so-called ‘*moody*’ strategy) wins, in that it maximally increases gains and limits resource losses in the long run. Since real ancestral environments were plausibly moderately predictable (Wilke & Barrett, 2009), this model gives us good reason to think that if the LMS did have such a function, it would improve fitness *in the way specified by the theory*. Moreover, the fact that low mood increases fitness in the forced swim test by helping mice save their energy further supports the propitiousness theory by showing that low mood, at least in these instances, increases fitness by limiting resource expenditure.

Furthermore, such a function isn’t carried out by other systems, thus avoiding the evolutionary irrelevance problem that affects the disease theory. While it’s true that other systems monitor current energy-levels and chance of success at an individual tasks, there is no other system that tracks overall chance of making net gains. Think about it this way: we know there is a system that monitors current energy levels—presumably the fatigue system. However, knowing current energy levels isn’t enough to know whether we stand to make net gains in a certain situation. To know that, we also need to integrate information about things like sickness, social status, interpersonal bonds, etc. The LMS is the system that does this.

## Potential Objections

### Extreme Circumstances

Despite the propitiousness theory’s ability to satisfy (i) and (ii), we cannot call it a day yet, as two potential objections remain. The first was formulated by Nettle (2009) and runs like this. In extreme circumstances, severely depressed humans and non-human animals manifest hyperactive, risk-prone, and impulsive behaviours. For example, severe food deprivation (an extremely unpropitious circumstance) leads animals to expend more energy than normal and take more risks, specifically by coming out of cover in the presence of predators. Nettle takes this as evidence that the LMS cannot merely function to limit resource expenditure by demotivating behaviour in relatively unpropitious circumstances. Rather, he proposes that low mood actually motivates behaviour in extremely unpropitious circumstances.

I argue that Nettle’s objection misses the point. It’s true that the propitiousness theory predicts that the LMS will *demotivate* organisms when there is little chance of them making net gains. But the fact that a system demotivates you to φ doesn’t mean that you will not φ—you might have a stronger, overriding motivation, issued by a different system. If we take this into account, we should see that the propitiousness theory predicts the data discussed by Nettle. Compare what happens in *moderately* vs *extremely* unpropitious circumstances. In the first case, we might be a bit hungry, but the not-too-strong signal “Eat!” is blocked by a stronger signal issued by LMS. In the second case, in contrast, we might be literally starving. Therefore, the motivation “Eat!” is likely to be so prepotent that it overrides the signal issued by LMS, leading to potentially risky foraging behaviour. I can put it like this. Nettle thinks that a theory of LMS should explain how low mood can *bring about* hyperactivity. This, I maintain, is a mistake. Humans and non-human animals in extremely unpropitious circumstances become hyperactive *in spite of* their low mood, not *because* of it. I conclude that Nettle’s objection doesn’t pose a real threat to Nesse’s theory.

### Subtypes of Low Mood

The propitiousness theory is a far more general theory than both the social risk theory and the disease theory. This may lead some to think that there are subtypes of low mood, and that the social risk and disease theories account for the proper function of the systems that underlie these subtypes. I’m sceptical, and have already laid out a number of arguments for why we should think there is a unified LMS in Sect. 2.1. However, given that the propitiousness theory’s generality may call into question whether the LMS is one system, I feel more should be said. In what follows, I argue that while more work needs to be done on discovering potential subtypes of low mood, we have good reason to think that the LMS nonetheless has a single proper function.

To show that the LMS is made up of several subsystems, each with their own proper function, I argue that one should do two things. Firstly, one should show that these subsystems are at least partially dissociable. E.g., if there is a “social risk subsystem” of the LMS, it should be the case that somebody should be unable to undergo a low mood following a social loss, while having the ability to experience low mood following, say, a personal failure.

Secondly, one should establish that ascribing a distinct proper function to each subsystem better explains the operation of the LMS. To this effect, one should look at the reliable, distal effects of low mood that arise in response to the situations that have the adaptive challenges posited by the theory in question. For example, in order to show that there is a social risk subsystem, one should look at cases of low mood that arise when social risk is high, and see if they cause behaviours that would a) lower social risk, and b) not be explained by the propitiousness theory alone. To illustrate, if there was a social risk subsystem, we would expect low mood that arises in response to situations with high social risk to improve hygiene, or at least have no effect on it, rather than make it worse. One would also have to show that responding in such a way in such circumstances would have increased fitness more so than if the LMS had a single proper function, as natural selection would not have endowed creatures with multiple adaptations that do nothing to increase fitness.

Do we have the kinds of evidence mentioned above? I argue not. There is little evidence of dissociation in supposed types of low mood, and although there is evidence that the specific symptoms of low mood differ under differing eliciting circumstances (Keller & Nesse, 2005), there is no evidence the low mood causes different behaviours that solve different adaptive problems in these scenarios. E.g., there is no evidence that social-risk-induced low mood improves hygiene, thus lowering social risk. Therefore, there is little positive evidence in favour of the “multiple subsystems hypothesis”. Moreover, we have reasons to believe that the LMS has a single proper function.

Firstly, low mood has similar effects under different circumstances, insofar as it has a global, demotivational effect on behaviour, *regardless of what causes it*—something that is not predicted by the “multiple subsystems hypothesis”. For instance, if there was both a disease subsystem and a social risk subsystem, we would expect cases of low mood that arise after social losses to not have an effect on personal activities, whereas cases that arise when one is ill would have such an effect. However, low mood has a *general*, demotivational effect on personal activities, even though causes of low mood vary widely, suggesting that there is no difference in such behaviour depending on the type of cause.

And secondly, evolution is a tinkerer and new adaptations are expensive and difficult to develop. Thus, one-system hypotheses should be preferred, *all things considered*, when giving an account of the evolution of any capacity. Why? Because new systems are costly and difficult to develop, and they will only be selected for when a) the relevant mutations occur as a matter of sheer chance (pre-selection) and b) the systems that result from these mutations offer a clear selective advantage to organisms with them (Darlington, 1977; Jacob, 1977). This of course does not close the book on this matter—more experiments should be conducted to assess behaviours that low mood causes in response to specific scenarios. For the time being, the propitiousness theory has the upper hand.

## Conclusion

I began this article by asking a vexed question in the evolutionary literature: what is the proper function of the low mood system? My answer has been that the proper function of the LMS is to limit resource expenditure in relatively unpropitious circumstances, exactly as hypothesised by the propitiousness theory. What does knowing this teach us about low mood? A great deal, it turns out.

Firstly, it can guide further research into how the LMS works—now that we know *what* the LMS does, we can start to assess *how* it does it. For example, we can move away from simply seeing what neural activations or neurochemical changes are associated with low mood in general, and begin to assess how different neural structures, say, detect that one’s situation has become unpropitious, or, say, how certain neurochemical changes demotivate behaviour in order to conserve resources.

Secondly, it explains why humans and other animals have the capacity for low mood at all—though a general feeling of being low may *seem* useless, it actually improved our ancestors’ fitness by helping them maintain their resources when resource expenditure would have likely been fruitless.

Thirdly, we now have a better understanding of the LMS’s normativity, and specifically how it can go wrong. We now know that cases of depression that arise with no distal cause whatsoever are dysfunctional because the LMS activates even though one’s chance of making net gains hasn’t changed. In hypomania, things are the other way around: the LMS fails to demotivate behaviour even when one’s situation becomes unpropitious.

Fourthly, the propitiousness theory potentially shines some light on the content of low mood. If the propitiousness theory is right, then low mood likely represents situational unpropitiousness, or that efforts in pursuits of gains are likely going to be wasted, or something similar (at least if we accept some form of teleosemantics) (Schulte & Neander, 2022).

Finally, knowing the proper function of the LMS may aid in the treatment of depression. A recent study suggests that framing depression as a useful signal, rather than as a disease, is helpful for patients’ recovery (Schroder et al., 2023). In my estimation, it would be fruitful to test if explaining what *exactly* low mood is useful for (i.e., explaining to patients what the proper function of the LMS is) has an even greater positive effect on patient outcomes than simply explaining that it has* a* function. If it does, then medical professionals should also seriously reconsider how depression is presented to the public, and perhaps refrain from referring to it as a dysfunction or a “chemical imbalance”, as it so commonly is (Horwitz & Wakefield, 2007).

## Footnotes

1 Other theories include the social competition theory (Price and Sloman 1987) and the analytical rumination theory (Andrews and Thompson 2009). I’m not discussing these theories here either because they have proven too weak in the face of several objections (see Hagen 2011), or because my arguments can be applied to those theories as well.

2 Thanks to an anonymous reviewer for Erkenntnis for suggesting I elaborate upon this point.

3 To clarify: by ‘low mood system’, both I and the evolutionary theorists cited are referring to a *functionally individuated* system—i.e., a cognitive system individuated in terms of the function it performs, rather than in terms of its physical implementation. (Allen and Badcock 2003; Kinney and Tanaka 2009; Nesse 2009).

4 These theorists think that *some* cases of depression will be activations of a dysfunctional LMS, but that these cases are rare.

5 What’s more, whether depression counts as a disorder is orthogonal to the purpose of this paper. All that matters for my arguments is that, regardless of its status as a disorder, we have good reason to believe that depression is the activation of a *properly function* LMS (which we do, as I outline in this section).

6 One exception is health, where their risk-taking (e.g., excessive smoking and drinking) is above average (Cobb-Clark et al., 2020). However, such behaviours are considered to be coping mechanisms for assuaging the unpleasantness of depression (Khantzian 1997), and so shouldn’t be considered when trying to determine the proper function of the LMS.

7 While failure to create art *could* affect one’s social risk in certain scenarios, there is plenty of evidence to suggest that creative activities affect low mood regardless of their impact on social risk (Brosowsky et al., 2022; Secker et al., 2018).

8 The term ‘net gains’ is my own, used as shorthand for Nesse’s notion of gains that are worth the investment needed to attain them.
